# Integrating Polymeric 3D-Printed Microneedles with Wearable Devices: Toward Smart and Personalized Healthcare Solutions

**DOI:** 10.3390/polym18010123

**Published:** 2025-12-31

**Authors:** Mahmood Razzaghi

**Affiliations:** Department of Mechanical Engineering, University of Victoria, Victoria, BC V8P 5C2, Canada; mahmoodrazzaghi@uvic.ca

**Keywords:** 3D printing, polymeric microneedle arrays, wearable devices, personalized healthcare, smart wearables

## Abstract

Wearable healthcare is shifting from passive tracking to active, closed-loop care by integrating polymeric three-dimensional (3D)-printed microneedle arrays (MNAs) with soft electronics and wireless modules. This review surveys the design, materials, and the manufacturing routes that enable skin-conformal MNA wearables for minimally invasive access to the interstitial fluid and precise but localized drug delivery. Looking ahead, the converging advances in multimaterial printing, nano/biofunctional coatings, and artificial intelligence (AI)-driven control are promising “wearable clinics” that can personalize monitoring and therapy in real time, thus accelerating the translation of MNA-integrated wearables from laboratory prototypes to clinically robust, patient-centric systems. Overall, this review identifies a clear transition from proof-of-concept MNA devices toward integrated, wearable, and closed-loop therapeutic platforms. Key challenges remain in scalable manufacturing, drug dose limitations, long-term stability, and regulatory translation. Addressing these gaps through advances in hollow MNA architectures, system integration, and standardized evaluation protocols is expected to accelerate clinical adoption. However, the realization of closed-loop wearable MNA-based systems remains constrained by challenges related to power consumption, real-time data latency, and the need for robust clinical validation.

## 1. Introduction

Over the last decade, wearable healthcare technologies have evolved from simple activity trackers into more multifunctional platforms that are capable of continuous physiological and even biochemical monitoring. The combination of microelectronics, flexible materials, and also wireless communication has turned wearables into quite a powerful tool for real-time health management and for more personalized medicine [1]. Modern devices include sensors that can track parameters like temperature, motion, heart rhythm, or even sweat composition, which makes possible preventive diagnostics and more decentralized care [2]. Altogether, this shift is showing a clear move from hospital-based monitoring toward more autonomous and patient-focused systems that are able to handle both data collection and closed-loop therapeutic actions.

Among the newer strategies in drug delivery and biosensing, microneedle arrays (MNAs) have received quite a lot of attention as minimally invasive systems for transdermal delivery and sampling [3,4]. By softly piercing through the stratum corneum without really triggering the pain receptors or causing any damage to the blood vessels, MNAs offer painless access to the interstitial fluid (ISF), which is a biomarker-rich medium that quite fairly reflects the blood composition [5,6]. This technology, in a way, is bridging the gap between invasive blood testing and non-invasive surface sensing, and it is enabling applications like continuous glucose tracking, lactate detection, and real-time metabolite monitoring [7,8]. The integration of MNAs with flexible electronics and also with wireless data transmission has led to the development of smart wearable biosensors that are able to perform multiplexed and real-time diagnostics [9,10]. These kinds of systems are already being used for chronic disease monitoring, personalized drug delivery, and even for on-skin health analysis, showing a good level of biocompatibility, mechanical stability, and also user comfort.

The intricate structure of MNAs has historically posed fabrication challenges [11]. The conventional fabrication methods like photolithography or molding are usually limited when it comes to scalability and geometric accuracy. Other techniques such as micromilling, lithography, and injection molding can work quite well, but they often require several complicated and costly steps, which again makes large-scale production rather more difficult [12,13,14,15]. On the other hand, three-dimensional (3D) printing offers unmatched design freedom and it allows engineers to adjust the microneedle geometry, density, and even the hollow structures based on specific anatomical or clinical requirements [11,16,17,18]. High-resolution 3D-printing techniques like stereolithography (SLA), digital light processing (DLP), and also two-photon polymerization (2PP) are now getting more and more optimized to produce complex microneedle structures with a higher precision and with much shorter fabrication times [19,20,21,22,23]. These developments are bringing the technology closer to clinical utility by enabling customizable MNA designs and also integration with other devices to control the function [24]. Recent works have been showing that 3D-printed MNAs can be incorporated with conductive pathways, microchannels, and bioresponsive coatings, which significantly enhance the versatility of wearable diagnostic and therapeutic platforms [25].

In addition, the emergence of affordable and high-resolution 3D printers has been greatly lowering both financial and technical barriers, promoting the wider use of MNA technology across research laboratories and also clinical environments [26,27]. This technique is also supporting the design of multifunctional MNAs by incorporating internal microchannels or reservoirs, which allow for controlled and localized drug delivery and open new possibilities for theranostic applications [26,28,29].

Materials development has been progressing in parallel with fabrication improvements. Biocompatible photopolymer resins and novel composites are now being used to create MNAs that can deliver challenging therapeutics. Polymers have emerged as the ideal materials for 3D-printed MNAs because of their biocompatibility, biodegradability, mechanical flexibility, and also chemical tunability [30]. Common polymers like poly(ethylene glycol) diacrylate (PEGDA), poly(lactic acid) (PLA), poly(ε-caprolactone) (PCL), poly(vinylpyrrolidone) (PVP), and gelatin methacryloyl (GelMA) can be engineered to obtain controlled stiffness and dissolution behaviors, allowing MNAs to dissolve, form hydrogels, or even act as drug-loaded reservoirs [31,32]. Polymer-based MNAs are also making possible some stimuli-responsive features, where they can react to things like temperature, pH, or different biochemical signals to provide on-demand drug release or a more dynamic sensing function [32].

The integration between materials development and improvements in fabrication methods is supporting real-time biosensing; for example, recent designs of hollow metallic microneedles are doubling as electrodes for glucose monitoring while also permitting insulin delivery [33]. Researchers have demonstrated the ability of 3D-printed MNAs to sample biological fluids safely from tiny volumes (e.g., the perilymph in the inner ear) [20], highlighting their potential in diagnostics.

Polymeric 3D-printed MNAs that are combined with wearable systems are now standing at the very front of smart and more personalized healthcare. They are bringing together the advantages of precise manufacturing, flexible biopolymers, and also digital connectivity to offer real-time monitoring, on-demand treatments, and adaptive feedback control. This meeting point between materials science, biomedical engineering, and additive manufacturing is likely reshaping the next generation of healthcare, leading the way toward more autonomous “wearable clinics” that can diagnose, treat, and also keep track of patients continuously in their daily life [5,10].

In this review, we are trying to give a broad overview about the recent advances in the design, fabrication, and also the biomedical integration of polymeric 3D-printed MNAs within wearable healthcare technologies. The focus is mainly on how additive manufacturing is providing more precise control over microneedle geometry, material choice, and also device architecture to reach a higher functionality, better patient comfort, and stronger personalization. The discussion covers the main 3D-printing methods, polymer materials, and also the different strategies for merging MNAs with flexible electronics, sensors, and wireless parts. Special attention is given to biomedical uses such as continuous glucose tracking, painless vaccination, and on-demand drug release, together with growing roles in multiplexed biosensing and physiological monitoring. The review is also about the ongoing challenges in scaling up production, clinical translation, and regulatory acceptance, while pointing to future directions that involve artificial intelligence (AI), self-powered systems, and closed-loop therapeutic setups on the road toward fully autonomous and personalized wearable healthcare.

**Figure 1:**
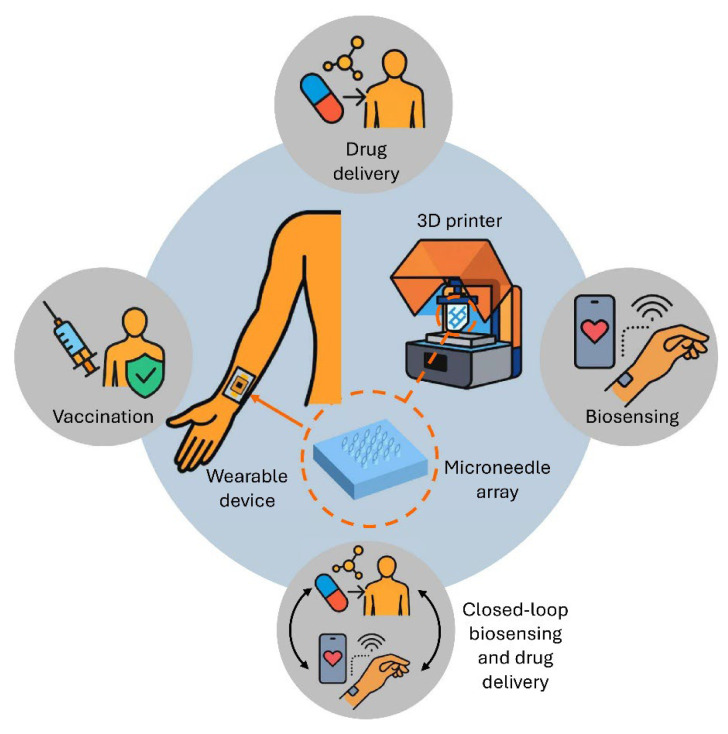
An illustration showing the different applications of 3D-printed MNAs integrated with wearable devices. The grey circular insets depict representative use cases (vaccination, drug delivery, biosensing, and closed-loop biosensing–drug delivery). The orange dashed circle highlights the MNA patch. The circular arrows denote the closed-loop feedback concept.

Although several reviews have examined 3D-printed MNAs or wearable biosensing technologies independently, most prior studies focus on fabrication methods, materials, or standalone device demonstrations without addressing system-level integration. In particular, the coupling of polymeric 3D-printed MNAs with soft electronics, wireless modules, and closed-loop therapeutic strategies remains fragmented across the literature. The present review fills this gap by providing a unified analysis of polymeric 3D-printed microneedle–wearable systems, emphasizing design strategies, fabrication scalability, sensing and drug-delivery integration, and translational challenges. By consolidating advances across biosensing, neuromuscular monitoring, and closed-loop therapy, this work offers a system-oriented perspective that is largely absent from existing reviews and highlights emerging opportunities toward smart and personalized healthcare platforms. Figure 1 shows the different applications of 3D-printed MNAs integrated with wearable devices.

## 2. Polymeric 3D-Printed Microneedle Arrays

This section provides an overview about the principles, benefits, and technological advances in the polymeric 3D printing of MNAs, with an emphasis on its advantages over conventional methods and also on its transformative impact toward wearable healthcare platforms.

### 2.1. Advantages and Emerging Applications of Microneedle Arrays

MNAs penetrate only the stratum corneum and the upper epidermis, avoiding the nerve endings and delivering a nearly pain-free experience. This characteristic makes MNAs particularly appealing for pediatric and needle-phobic populations [34,35,36]. MNAs deliver drugs directly into the systemic circulation, ensuring higher bioavailability and faster onset of the action. This property is especially beneficial for biologics such as peptides and proteins, which are susceptible to enzymatic degradation inside the gastrointestinal tract [35,37,38].

Patient compliance is another area where MNAs are really excelling. Their ease of use allows self-administration, which reduce the need for trained healthcare professionals. This feature is particularly advantageous for chronic conditions that require frequent drug administration, such as diabetes or hypertension. MNAs also eliminate the risks related to needle-stick injuries, reducing the burden from medical waste and improving overall safety [39,40,41].

The precision and the control that are offered by MNAs further distinguish them from traditional methods. MNAs are able to deliver drugs in a more localized manner, which minimizes systemic exposure and reduces the risk of side effects. Additionally, their ability to incorporate controlled-release mechanisms allows sustained drug delivery over time, reducing the frequency of administration and improving therapeutic outcomes. For instance, dissolving MNAs that are made from biodegradable materials can release drugs gradually as they dissolve inside the skin, which ensures a steady therapeutic effect [35,36,41].

Beyond their medical applications, MNAs are also gaining more traction in the cosmetic industry. They are used for antiaging treatments, skin rejuvenation, and also for the delivery of active ingredients in skincare products. The ability to target the dermal layers without causing significant discomfort has made MNAs a quite popular choice for cosmetic enhancements [35,40,42].

As MNA technology is continuing to evolve, its potential applications are expanding more into diagnostics and also personalized medicine. Recent innovations include MN-based biosensors that are capable of monitoring biomarkers in real time. These kinds of devices provide a minimally invasive alternative for continuous health monitoring, offering valuable insights about a patient’s physiological state. The integration of MNAs with wearable technologies is further enhancing their utility, enabling point-of-care diagnostics and also remote healthcare management [26,36,41,43].

**Figure 2:**
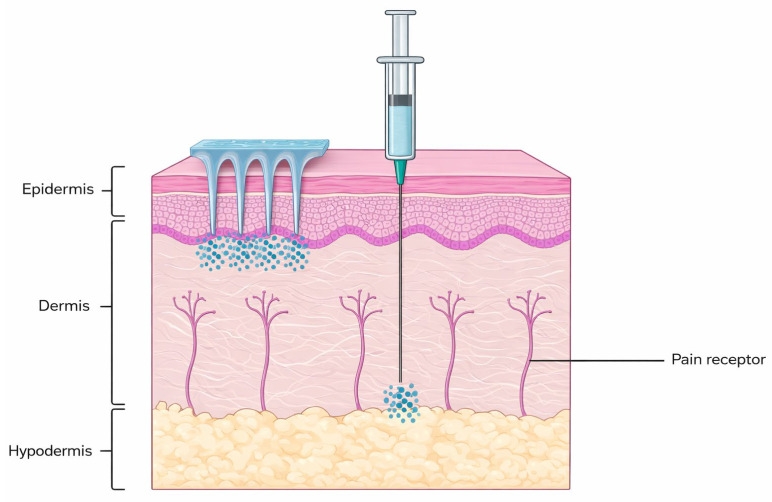
A comparison between conventional hypodermic needle injection and MNA delivery. The epidermis (pink), dermis (light pink), and hypodermis (yellow) are shown schematically. The MNAs (blue) penetrate only through the stratum corneum and the upper epidermis, avoiding the nerve endings while efficiently enabling the transdermal transport of the drugs (blue dots) into the systemic circulation. Sensory nerve endings/pain receptors are illustrated as purple fibers and labeled. This minimally invasive mechanism allows their painless administration, high patient compliance, and also improved bioavailability. Reprinted under a CC BY license [44].

Figure 2 shows a comparison between conventional hypodermic needle injection and MNA delivery.

### 2.2. Three-Dimensional-Printing Techniques for Polymeric MNAs

Several 3D-printing techniques have been adapted for the production of MNAs, and each one offers unique advantages that are suited for specific applications [45,46]. Among the diverse 3D-printing techniques, SLA, DLP, 2PP, and also fused deposition modeling (FDM) are some of the most widely utilized methods for fabricating MNAs. SLA is among the most widely adopted 3D-printing techniques to fabricate MNAs. This method employs an ultraviolet (UV) laser to selectively cure liquid photopolymers into precise solid structures. SLA offers high resolution and smooth surface finishes [47]. It is particularly well-suited for creating intricate MN designs. Its versatility has led to extensive use in biomedical applications, including the development of advanced drug-delivery systems and diagnostic tools, where the precision and the surface quality are quite critical [17,45]. SLA has been extensively employed for the 3D printing of MNAs [13,47,48,49,50,51,52,53,54,55,56,57]. It has enabled the fabrication of various types of MNAs, including hollow, dissolvable, and also solid forms, for transdermal drug-delivery applications [54,55,56,58]. Also, this technology is being utilized to fabricate master molds for dissolvable MNAs that are designed for ocular drug delivery [52], as well as for coated MNAs that are used for intradermal insulin administration [53].

Despite their high resolution and design flexibility, 3D-printing techniques such as SLA, DLP, and 2PP present important limitations that must be considered for MNA fabrication and clinical translation. Print anisotropy arising from layer-by-layer photopolymerization can lead to direction-dependent mechanical properties, potentially affecting MNA insertion reliability and fracture resistance. In addition, oxygen inhibition during free-radical photopolymerization may compromise surface curing fidelity, particularly for fine tip geometries [59]. From a biocompatibility perspective, residual photoinitiators and unreacted monomers in photopolymer resins raise concerns regarding cytotoxicity, necessitating extensive post-processing, solvent extraction, or surface coating strategies [60]. These limitations highlight the need for careful material selection, optimized curing protocols, and standardized biocompatibility assessments when translating 3D-printed MNAs toward wearable clinical applications.

SLA-printed MNAs have been employed in some innovative applications such as for transdermal electrochemical sensing [61] and also as the base substrates for lab-on-a-MNA systems that are capable of rapid biomarker detection in finger-prick blood samples. They have been also used for blood-free detection of biomarkers such as C-reactive protein and procalcitonin in ISF [13].

DLP is another widely used 3D-printing technique for fabricating MNAs. As a variation of SLA, DLP improves production efficiency by curing entire layers of photopolymer resin simultaneously through using a digital light projector [62]. In other words, unlike SLA 3D printing where the resin is cured point by point, DLP technology projects light across the entire layer at once by using a projector. This approach enables the rapid curing of each layer, significantly accelerating the printing process [63,64]. The DLP technique has been extensively utilized for the 3D printing of MNAs [15,26,57,65,66,67,68,69,70]. This method offers a relatively high resolution, typically reaching the micron scale [71]. DLP-printed MNAs that are fabricated from polyethylene glycol diacrylate (PEGDA) have been designed for applications such as on-demand drug delivery and the multiplexed detection of biomarkers, including pH, glucose, and also lactate levels in the skin ISF [26]. Additionally, continuous glucose monitoring in the ISF has been successfully achieved by using solid MNAs that are produced with biocompatible and light-sensitive resins, as was demonstrated through in vivo testing on mice [67]. Moreover, DLP has been facilitating the development of drug-delivery systems that employ MNAs made from biocompatible resins, which enable increased permeability of active compounds with molecular weights ranging between 600 and 4000 Da in the buccal tissue [15].

The liquid crystal display (LCD) technique is another process that is being employed for the 3D printing of MNAs [56,72,73,74]. Like SLA and DLP, the LCD technique is also a vat photopolymerization process that uses light-curable resins to fabricate highly precise microstructures such as MNAs. However, its key advantage is offering a comparable resolution but at a much lower cost, which makes it particularly suitable for the large-scale manufacturing of complex designs while still maintaining both the accuracy and the cost-effectiveness [75]. Like DLP, the LCD-based printing technique offers higher printing speeds than SLA, since it cures each layer in a single exposure instead of scanning point by point. The key distinction between the LCD technique and DLP is the way the light is projected. In DLP printing, a digital micromirror device projects the full image of each layer onto the resin surface, whereas LCD printing relies on a liquid crystal display screen that selectively transmits the light through individual pixels to solidify the resin layer by layer. This pixel-controlled illumination minimizes image distortion and enhances the uniformity, although it generally results in a slightly longer curing time compared with DLP systems [76].

Compared with SLA and DLP techniques, the LCD technique typically offers comparable resolution, in the range of ~30–100 μm [77]. In LCDs, the achievable curing depth is typically limited by the relatively low ultraviolet transmission efficiency and contrast of the LCD mask, which reduces effective irradiance and necessitates longer exposure times to achieve sufficient interlayer bonding. Compared with DLP systems that employ high-intensity projected light, LCD printers generally exhibit slower build speeds despite curing entire layers simultaneously, owing to reduced light intensity and increased optical diffusion through the pixelated mask. While these factors constrain resolution and curing efficiency, LCD printing remains an attractive platform for MNA fabrication due to its low system cost, high level of commercialization, and adequate resolution for many polymeric MNA designs, particularly in scalable and cost-sensitive wearable applications [78].

Beyond fabrication resolution, the mechanical performance of MNAs is a critical determinant of successful skin penetration and device reliability. SLA and DLP-fabricated MNAs typically achieve smooth surfaces and moderate tip radii, resulting in reliable insertion forces but mechanical strength that is sensitive to print orientation and post-curing conditions. LCD-printed MNAs generally exhibit larger effective tip radii due to pixelation effects, which can increase insertion force and reduce penetration efficiency [78]. In contrast, 2PP enables submicron tip radii and highly uniform geometries, yielding low insertion forces but often limited fracture resistance due to slender aspect ratios and material constraints. These trade-offs highlight the need to balance geometric sharpness with mechanical robustness when selecting fabrication routes for wearable MNA applications.

Static optical projection lithography (SOPL) is another emerging 3D-printing technique that is being applied for the fabrication of MNAs. In this approach, a patterned light field is statically projected to initiate the polymerization inside the monomer solution according to the spatial distribution of the light intensity. This process allows for rapid MNA fabrication without the need for any mechanical movement, achieving exceptionally high throughput. Moreover, by just modifying the projected images, SOPL can produce MNAs with diverse geometries and also MNAs with the structural variations [17]. Furthermore, SOPL-fabricated MNAs avoid the layer-by-layer structure typical of 3D-printing methods, such as DLP, and showed a fracture force of about 4 N in compression testing, indicating sufficient mechanical strength for skin insertion [19]. This technique enables highly precise fabrication for microneedles with intricate geometries and allows rapid design customization. The resulting MNAs feature smooth surface morphologies that minimize insertion-induced tissue damage and enhanced overall biocompatibility [19].

Recent advancements in 3D printing include 2PP, which enables nanoscale precision. The 2PP technology can create MNAs with extremely sharp tips and also intricate internal features. Although 2PP is costly and time-consuming, its ability to produce high-resolution structures makes it a quite promising choice for research and also for specialized applications [17,20].

The 2PP technology uses ultrashort laser pulses from a near-infrared femtosecond laser to selectively polymerize photosensitive resins. This process involves the nearly simultaneous absorption of two photons, which generates electronic excitation that is equivalent to the one produced by a single photon with higher energy. This absorption results in non-linear energy distribution that is focused precisely at the laser’s focal point, with only minimal absorption outside of the immediate focal volume. When this energy is absorbed, the photoinitiator molecules inside the resin initiate the polymerization process within localized areas known as the “polymerization voxels,” where the energy exceeds a specific threshold. Compared with other techniques, 2PP offers superior geometry control and a scalable resolution, while also reducing the costs that are associated with equipment, facilities, and maintenance in etching and lithography-based methods. This has made 2PP a quite versatile tool for fabricating solid and also MNAs by using materials such as modified ceramics, inorganic–organic hybrid polymers, acrylate-based polymers, polyethylene glycol, and more recently water-soluble materials, with highly promising results [79].

One of the key advantages of 2PP is its ability to achieve resolutions as fine as about 100 nm [80]. Researchers have been employing 2PP to mold dissolving and also hydrogel-forming MNAs by using aqueous blends of PVP and poly(vinyl alcohol) (PVA) for controlled drug delivery through skin models [79]. It has also been used to fabricate MNAs from organically modified ceramic hybrid materials (Ormocer^®^, Fraunhofer-Gesellschaft, Würzburg, Germany) for transdermal drug delivery [81]. These MNAs exhibit excellent mechanical stability, with no breakage and with minimal or almost no bending at the tip after surgical use [82].

Additionally, a hybrid method that combines 2PP with electrochemical deposition has been developed to create ultrasharp, gold-coated copper solid MNAs for inner ear drug delivery [83]. Furthermore, 2PP has been utilized to produce MNAs that are designed for the transdermal sensing of electrolytes, such as potassium (K^+^) ions [84].

FDM is another method that can be used for the 3D printing of MNAs. In FDM, thermoplastic filaments are melted and extruded through a heated nozzle to build structures layer by layer. While this method is economical and simple, its resolution is quite limited, which makes it less suitable for creating the fine details that are required for MNAs [85,86]. Despite this, the advances in FDM technology have enabled its use for the prototyping of MNAs with relatively simple geometries. Also, although its resolution is lower compared with other 3D-printing methods, post-processing techniques like chemical etching can improve its utility for MN fabrication. FDM is particularly suitable for biodegradable MNAs [87].

FDM has been utilized in several studies to produce MNAs [88,89,90,91]. This 3D-printing technique is widely favored because of its rapid production, cost-effectiveness, easy accessibility, and also versatility in material usage [92]. However, post-processing plays a quite critical role in FDM, since the printed components are not immediately ready for use. Once the printing is complete, the product is removed from the print bed, any supporting structures are detached, and the item undergoes post-processing to enhance its surface quality [93]. In the realm of the 3D printing of MNAs, some innovative approaches have successfully combined FDM with post-fabrication etching techniques to create needles having the optimal size and shape. These kinds of needles can penetrate into the skin, break off, and deliver small molecules without requiring any master template or mold [88]. Additionally, coated polylactic acid (PLA) MNAs have been developed for effective transdermal drug delivery [91].

**Figure 3:**
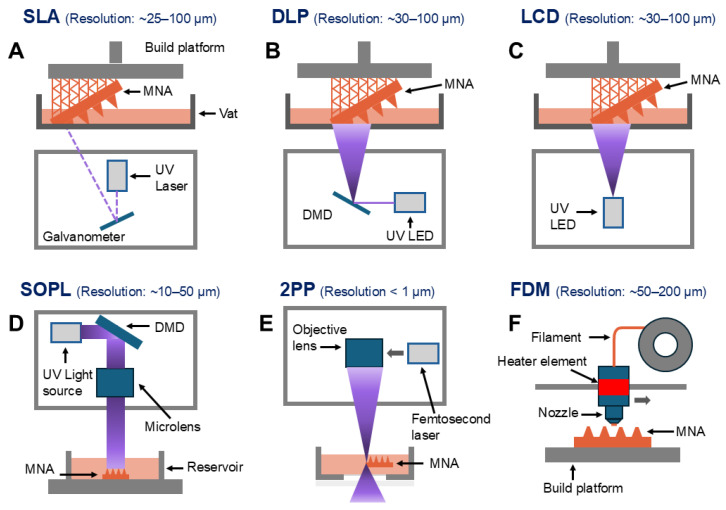
Schematic representation of the working principles of the main 3D-printing techniques utilized for polymeric MNA fabrication: (**A**) SLA, (**B**) DLP, (**C**) LCD, (**D**) SOPL, (**E**) 2PP, (**F**) FDM. In all schematics, colored beams indicate the illumination path used for photopolymerization. Colors are used only for visual distinction of components (light source/optics, polymer resin/filament, and printed MNA) and do not represent quantitative values. Reprinted under a CC BY license [77].

Figure 3 schematically illustrates the working principles of key 3D-printing techniques that are used for polymeric MNA fabrication, including SLA, DLP, LCD, SOPL, 2PP, and also FDM. Also, Table 1 represents an overview of MNA types, technologies, costs, and application fields. Furthermore, Table 2 represents a comparison of MNA 3D-printing techniques, materials, achievable dimensions, strengths, limitations, and technology readiness levels (TRLs).

Although 3D-printing techniques such as SLA and DLP offer high design flexibility and reproducibility, their direct fabrication of microneedles is typically limited by optical resolution, pixelation, and light scattering, resulting in practical tip sizes generally above 50 µm. To address this limitation, recent studies have increasingly adopted hybrid fabrication strategies in which 3D-printed MNAs serve as high-precision master molds rather than final devices. Lijnse et al. [94] demonstrated that low-cost DLP printing can be effectively used to fabricate conical microneedle master molds with controlled geometry, which were subsequently replicated through elastomeric molding to produce polymer microneedles with reduced tip dimensions suitable for biomedical applications. Similarly, Justyna et al. [95] employed DLP-printed master molds combined with replica micromolding to fabricate dissolving microneedles with tip diameters below 50 µm, achieving reliable skin insertion, sufficient mechanical strength, and rapid dissolution profiles for drug-delivery applications. These studies highlight that, while direct 3D printing alone may not consistently achieve sub-50 µm microneedle tips, the use of 3D-printed master molds followed by secondary molding processes effectively bridges this resolution gap. This hybrid approach preserves the advantages of additive manufacturing, such as rapid design iteration and customization, while enabling the fabrication of high-performance microneedles with sharper tips and improved functional reliability.

### 2.3. Material Selection for 3D Printing of Microneedle Arrays

The selection of materials is a quite crucial aspect in the 3D printing of MNAs, as it directly affects their mechanical properties, biocompatibility, and also the overall functionality. Polymers, ceramics, and metals are the primary materials that are used, and each one is chosen based on the intended application. Polymers are the most commonly employed materials because of their versatility and good biocompatibility. PLA, polycaprolactone (PCL), and also PVA are popular choices for creating biodegradable and dissolvable MNAs. These materials are ideal for drug-delivery applications, since they can encapsulate the active pharmaceutical ingredients and release them gradually while the MNAs are dissolving in the skin [96,97]. Hydrogels, which are water-absorbing polymers, are another important class of materials. They are used to create hydrogel-forming MNAs that swell upon contact with skin fluids, enabling sustained drug release or biosensing capabilities [18]. Despite their promising performance, hydrogel-forming MNAs also present limitations that must be considered for prolonged or wearable use. Because drug transport relies on continuous hydration and swelling of the polymer network, extended wear may lead to partial dehydration under low-humidity or non-occlusive conditions, potentially altering diffusion-controlled release kinetics. Moreover, prolonged occlusive contact with the skin may increase the risk of microbial growth at the skin–device interface, particularly in the absence of integrated antimicrobial strategies. Although it has shown favorable skin recovery, intact removal, and acceptable tolerability up to 24 h following application in human volunteers, longer-term clinical validation remains limited. These factors highlight the need for careful material selection, hydration control, and infection-mitigation strategies when translating hydrogel-forming MNAs to extended-wear or wearable therapeutic systems [98].

Ceramics offer superior mechanical strength and are often used in applications that require durable and reusable MNAs. However, the brittleness of ceramics poses some challenges in their fabrication and also during application [20,99]. Metals, such as stainless steel, are well known for their durability and mechanical robustness [100].

Hybrid materials and composites have been gaining attention because of their ability to combine the desirable properties of different materials. For instance, polymer–ceramic composites can offer the flexibility of polymers together with the strength of ceramics [81]. Advances in bioinks that are specifically designed for the SLA, DLP, and also 2PP processes have been further expanding the range of the materials that are available for MN fabrication, allowing the direct integration of drugs or biologically active agents inside the MN structure [17,20].

Hydrogels are a vital category that swell upon contact with skin fluids to deliver drugs or to facilitate biosensing. The photopolymers that are used in the SLA, LCD, and DLP processes are valued because of their ability to produce high-resolution MNAs, but they require careful selection to ensure biocompatibility [62]. According to ISO 10993 standards, materials intended for prolonged skin exposure should be assessed for cytotoxicity (ISO 10993-5) [101], skin irritation and sensitization (ISO 10993-10) [102], and, where applicable, chemical characterization and leachables (ISO 10993-18) [103]. Several commercial photopolymer resins have demonstrated acceptable short-term cytocompatibility following extensive post-curing and solvent extraction; however, residual photoinitiators and unreacted monomers remain a concern for chronic or repeated wear. These considerations highlight the need for standardized biocompatibility testing and resin-specific validation when translating photopolymer-based MNAs to wearable clinical applications. Polymer materials used in vat-photopolymerization-based MNA fabrication are subject to time-dependent degradation that can affect long-term wearable performance. Prolonged exposure to UV light during printing and post-curing may induce chain scission, residual crosslinking gradients, and embrittlement, while environmental factors such as humidity and temperature accelerate aging and property drift. In addition, the layer-by-layer curing inherent to vat photopolymerization can introduce anisotropy and interlayer weakness, making printed polymers susceptible to mechanical fatigue under repeated deformation or cyclic loading. These effects highlight the need for careful resin selection, optimized curing protocols, and durability testing when MNAs are intended for repeated or prolonged wearable use [78].

Table 3 provides a comparative overview of representative polymers used in microneedle array fabrication, summarizing their mechanical moduli, swelling behavior, degradation characteristics, and suitability for drug loading, to highlight key material trade-offs relevant to wearable applications.

### 2.4. Computational Modeling and Simulation-Guided Design of Microneedle Arrays

Computational modeling has become an important tool for guiding the rational design and optimization of MNAs, particularly when experimental characterization of skin–device interactions is challenging or resource-intensive. Among available approaches, finite element analysis (FEA) is most widely used to evaluate mechanical performance, insertion behavior, and failure risks prior to fabrication. Recent studies have shown that FEA can reliably predict insertion force, stress distribution, buckling, and penetration depth as functions of microneedle geometry, material properties, and array configuration [104].

Mechanical FEA models commonly represent skin as a multilayer structure comprising the stratum corneum, epidermis, and dermis with distinct elastic or hyperelastic properties. Using this framework, Wang et al. demonstrated that array spacing strongly influences insertion force and penetration depth, with diminishing performance gains beyond center-to-center distances of ~500–600 μm, providing quantitative guidance for array design [104]. Similarly, Yolai et al. [105] employed three-dimensional FEA to optimize microneedle geometry and array arrangement for transdermal vaccine delivery, showing that non-square array configurations can enhance delivery efficiency by improving interactions with dermal antigen-presenting cells.

Beyond insertion mechanics, advanced modeling frameworks increasingly integrate diffusion and transport simulations to predict drug or antigen release kinetics following microneedle insertion. Coupled diffusion–reaction models have been used to compare coated and dissolving microneedle architectures and support dose optimization without extensive in vivo testing [105]. Computational studies have also highlighted the impact of physiological variability. Azarikhah et al. [106] incorporated age-dependent skin properties into FEA models, revealing increased insertion force requirements in older skin due to changes in stratum corneum stiffness. Finally, simulation-based analyses of off-axis insertion and skin curvature have shown that small misalignment angles can induce stresses exceeding polymer yield strength, emphasizing the importance of geometry and alignment for reliable wearable MNA performance [105].

While finite element modeling provides valuable insights into MNA mechanics and skin–device interactions, model accuracy ultimately depends on validation against experimental data. Several studies, emphasizing the importance of calibrating simulation parameters using experimental results to ensure predictive reliability that have been benchmarked experimentally, show good correlation between predicted and measured insertion or extraction mechanics. For example, experimentally validated mathematical models have demonstrated strong consistency between insertion/extraction forces and simulation outputs in hollow MNA systems, supporting the credibility of numerically derived mechanical insights. However, systematic validation across standardized testing conditions and diverse skin types remains limited. This highlights the importance of integrating experimental benchmarking with computational modeling to improve predictive reliability and support translation of the MNA designs to wearable applications [107,108,109].

## 3. Integration of Microneedle Arrays with Wearable Devices

This section explores the design principles, materials, and technologies that are enabling the seamless integration of MNAs with wearable platforms, focusing on skin conformality, sensor coupling, wireless data transfer, and also user-centered functionality. The recent progress in 3D printing has been providing versatile methods for fabricating hollow and solid MNAs that are tailored for continuous contact with the skin. The layer-by-layer customization in the 3D-printing process enables precise control of the dimensions and the architectures that are critical for secure and painless skin penetration [45].

Material innovations are also playing a crucial role in enhancing skin adherence and conformality. Keirouz et al. [110] have reported a conductive polymer-coated 3D-printed MNA, allowing repeated skin insertions without losing conductivity or structural integrity. Economidou and colleagues [54] have further advanced this by integrating hollow MNAs with microelectromechanical systems (MEMSs), enabling controllable and personalized drug delivery while maintaining stable contact on the skin tissue. Also, Zhang and colleagues [111] have introduced a high-sensitivity hydrogel-based MNA wearable sensor that was fabricated through DLP 3D printing, which was designed to conform to joint surfaces and to capture subtle biomechanical deformations, illustrating the adaptability of MNAs for complex anatomical sites.

Beyond material flexibility, stimuli-responsive designs are being increasingly explored to improve conformal wear and responsiveness toward physiological conditions. Smart MNAs that are composed of stimuli-responsive polymers can adjust their functionality according to environmental triggers, providing controlled biomarker detection and on-demand therapeutic release [32]. Multifunctional MNAs can integrate biosensors, microfluidics, and smart biomaterials, thereby enabling closed-loop systems that are continuously attached on the skin while autonomously managing biosensing and drug delivery [112]. The responsiveness kinetics of stimuli-responsive MNAs vary significantly with material composition and physiological conditions. Hydrogel-based MNAs typically exhibit swelling times ranging from seconds to several minutes, with drug-release rates strongly influenced by pH, ionic strength, and local temperature. In glucose- or enzyme-responsive systems, release slopes depend on analyte concentration and diffusion kinetics, leading to variable response times under fluctuating physiological conditions [113].

The integration into personalized healthcare workflows demonstrates the translational promise of skin-conformal MN platforms. Yang et al. have introduced an MNA-based continuous biomarker and drug monitoring system enabling real-time pharmacokinetic and pharmacodynamic evaluations in diabetic patients, seamlessly connecting skin-conformal MNA sensors with smartphone interfaces to provide continuous clinical feedback [114]. This study illustrated how skin-conformal MNA platforms not only improve contact stability but also form the foundation for precision medicine systems that are capable of real-time therapeutic adjustment.

From a system-level perspective, MNA-integrated wearable platforms can be broadly classified into four functional categories: passive, semi-active, active, and closed-loop systems. Passive platforms rely on MNAs for skin access or sustained release without external control, while semi-active systems incorporate basic electronics for data acquisition or timed actuation. Active systems enable externally triggered or programmable sensing and delivery through integrated power, wireless communication, or microfluidic control. Fully closed-loop platforms combine real-time biosensing with automated therapeutic response, representing the most advanced and clinically relevant architecture for personalized healthcare.

Recent innovations demonstrate the effectiveness of MNAs in merging biosensing with flexible electronic substrates. Zhan et al. [115] have developed a 3D-printed hollow-MNA-based extraction system that was integrated with patterned electrodes, achieving minimally invasive monitoring of analytes such as glucose, pH, and hydrogen peroxide. In another study, our group has presented a remotely controlled theranostic platform that was based on hollow MNAs embedded with colorimetric and electronic sensors, which was coupled to a smartphone interface for continuous health monitoring and on-demand drug delivery. This system showcased the potential of flexible and portable electronics to democratize healthcare access across geographic and socioeconomic barriers [26]. Also, Rezapour Sarabi and colleagues [116] have shown how 3D printing is enabling the precise fabrication of MNA-based biosensing patches, which are compatible with polymeric and inorganic materials, to extract and analyze ISF.

Flexible electronics also underpin the emergence of MNA-based real-time biosensors for continuous clinical applications. Flexible and miniaturized designs support closed-loop systems, which integrate biosensing with therapeutic delivery, offering a new paradigm for personalized medicine [9].

Smart multifunctional MNA platforms are increasingly incorporating wireless modules to link biosensing and drug-delivery systems with external devices. Despite these advances, some challenges remain in minimizing power consumption, ensuring secure data transfer, and maintaining signal stability during continuous skin contact. In addition to power constraints, data transmission reliability remains a critical challenge for wireless MNA-based wearable systems, particularly in closed-loop healthcare applications. Wireless body area networks commonly experience packet loss, communication latency, and limited effective sampling rates due to contention-based medium access, interference, and dynamic body motion. These effects can degrade the timely delivery of physiological data and compromise real-time feedback control, especially for delay-sensitive or emergency signals. The use of low-power communication protocols further exacerbates these issues by imposing bandwidth and duty-cycle limitations. Research emphasizes the need for transmission prioritization, buffering strategies, and fail-safe mechanisms to ensure reliable data flow in closed-loop and safety-critical wearable systems [117].

Also, wearable health monitoring devices are particularly vulnerable to security threats arising from wireless data transmission and cloud-based data management, including unauthorized access, data interception, and integrity violations. Such risks are amplified by the continuous collection and transmission of sensitive physiological and medical data, which, if compromised, may directly affect patient safety and clinical decision making. Authors further emphasize that compliance with healthcare data protection requirements necessitates secure communication channels, robust authentication mechanisms, data integrity verification, and controlled access to cloud servers. These considerations are especially important for closed-loop and intelligent wearable systems, where the confidentiality, integrity, and availability of data are essential for safe and reliable operation [118].

While AI presents opportunities for adaptive control in closed-loop medical systems, AI/ML-based software faces significant challenges related to data quality, model validation, and transparency. The training and testing datasets must be representative, well-characterized, and sufficient to support reliable performance across diverse patient populations and use conditions, which can be difficult for systems relying on individualized physiological data streams. Robust validation, ongoing performance monitoring, and predefined change management plans are therefore required to ensure safety and effectiveness throughout the product lifecycle. In addition, algorithm transparency and interpretability are important to support clinical trust, risk management, and regulatory oversight, particularly for safety-critical applications involving automated or adaptive therapeutic decision making [119].

Self-powered technologies offer a quite promising alternative. Liang et al. [120] have developed a self-powered hydration-monitoring and drug-delivery patch to treat atopic dermatitis, integrating a piezoelectric generator to harvest biomechanical energy from body movements. This system operated autonomously, combining hydration sensing with on-demand MNA drug release in a closed-loop format, thereby eliminating reliance on external batteries.

Wireless energy transfer is another emerging strategy. Zhang et al. [121] have introduced a wirelessly powered near-infrared light MNA device that was designed for synergistic wound healing. Their platform integrated drug-loaded MNAs with a near-infrared (NIR) light-emitting diode (LED) array that was powered through electromagnetic induction, enabling stable energy delivery without bulky power sources. By combining phototherapy with localized drug release, the system demonstrated how wireless powering can expand therapeutic capabilities while maintaining device thinness and flexibility.

Energy harvesting from motion or body heat, when it is combined with wireless charging through near-field communication (NFC) or inductive coupling, provides a pathway toward autonomous and patient-friendly systems that are capable of continuous health monitoring [122].

One effective strategy is combining a rigid MNA with soft and flexible substrates. Rajabi et al. [123] have demonstrated flexible and stretchable MNAs integrating stainless steel MNAs into an elastomeric base. This design allowed the patch to adapt to skin wrinkles and movements while still ensuring painless penetration, illustrating how hybrid material selection enhances both functionality and comfort.

Integrating rigid MN structures with soft wearable substrates requires robust interfacial adhesion to accommodate mechanical mismatch and repeated deformation. Common strategies include mechanical interlocking at the MN base, elastomeric interlayers to reduce modulus mismatch, and surface modification techniques such as plasma treatment or silane coupling to enhance bonding. Hybrid fabrication approaches combining rigid MN cores with soft overmolded substrates have shown improved durability and wearer comfort in wearable systems [124].

Beyond initial mechanical compliance, the long-term performance of soft–rigid hybrid MNA patches is governed by fatigue behavior under repeated bending, stretching, and skin motion. Cyclic loading can induce interfacial debonding, crack initiation at stiffness transitions, and gradual degradation of insertion reliability. Consequently, repeat-cycle durability testing and strain-mitigating designs are essential to ensure stable performance during extended wearable use.

Bioadhesive and hydrogel-based platforms are another important design pathway. Xue et al. [125] have developed a wearable and flexible ultrasound MNA incorporating a bioadhesive hydrogel elastomer, ensuring a robust adhesion on curved and dynamic skin surfaces and minimizing detachment during motion.

User comfort is also linked with system ergonomics and device usability. Economidou et al. have demonstrated a 3D-printed hollow MNA MEMS device that was capable of precise and personalized delivery, showing how ergonomic designs can reduce treatment complexity and also enhance patient adherence [54].

As wearable MNA platforms increasingly integrate wireless connectivity and cloud-based analytics, interoperability and data security emerge as critical challenges. Heterogeneous hardware, communication protocols, and data formats can limit seamless integration across devices and healthcare systems. In parallel, continuous physiological data transmission raises concerns regarding data privacy, cybersecurity, and regulatory compliance. Addressing these issues through standardized communication frameworks, secure data encryption, and privacy-by-design architectures is essential for the safe clinical adoption of connected MNA-based wearables.

Quantitatively, wireless powering approaches used in wearable MNA platforms typically harvest power in the µW–mW range, depending on the mechanism (e.g., inductive coupling, RF, or energy harvesting). These levels are sufficient for low-power sensing, control electronics, and threshold-based actuation but remain limited for sustained pressure-driven drug delivery, which may require transient mW-level power. Consequently, many MNA systems employ intermittent actuation, energy storage, or hybrid powering strategies to balance harvested power with delivery energy demands.

Recent work shows that integrated power sources can better meet the energy demands of drug-delivery-intensive wearable MNA systems than wireless harvesting alone. Zhou et al. [126] demonstrated a magnesium-battery-powered iontophoresis patch providing stable current densities (10–100 µA cm^−2^) and sufficient energy density (~3.6 mWh cm^−2^) for controlled transdermal drug delivery, highlighting the limitations of low-power wireless harvesting for sustained actuation.

## 4. Biomedical Applications of MNA-Integrated Wearables

### 4.1. Biosensing and Continous Health Monitoring

Different studies have investigated using MNA-based wearable devices for biosensing and health monitoring. For instance, Parrilla et al. [127] have demonstrated a 3D-printed hollow MNA potentiometric platform that was functionalized with conductive inks, validating its ability to sample ISF and to detect biochemical analytes on the skin in a minimally invasive manner. In another study, Zhan et al. [115] have extended this concept by integrating 3D-printed hollow MNAs with patterned electrodes, enabling electrochemical detection for glucose along with other metabolites. Their system demonstrated accurate glucose detection both in vitro and in vivo, highlighting the potential of MNAs for minimally invasive and continuous health monitoring.

Also, Zhou et al. [128] have developed a 3D-printed MNA for real-time and painless glucose monitoring in interstitial fluid. Biocompatible resin needles (400 µm base, 1200 µm height) were coated with Au/Prussian Blue (PB)/glucose oxidase (GOD) and Ag/AgCl electrodes for selective enzymatic glucose detection. The compact circular layout minimized insertion force and improved wearer comfort. Electrochemical tests demonstrated high sensitivity (3.5 nA mM^−1^), strong linearity (R^2^ > 0.96), and excellent selectivity against interfering metabolites, indicating a low-cost and wearable platform for continuous diabetes management.

For electrochemical MNA-based biosensors, long-term performance is strongly influenced by electrode impedance stability, signal drift, and calibration requirements. Changes in electrode–tissue interface impedance over time, biofouling, and mechanical micromotions can lead to baseline drift and reduced sensitivity during prolonged wear. Consequently, periodic recalibration, reference electrode stabilization, and surface modification strategies are often required to maintain reliable signal fidelity in wearable and continuous monitoring applications.

During extended wear, MNA surfaces are susceptible to biological fouling, including protein adsorption and biofilm formation, which can compromise sensing accuracy and long-term device reliability. Adsorption of proteins and biomolecules from interstitial fluid or sweat may alter electrode surfaces, increase impedance, and induce signal drift in electrochemical sensors, as reported for wearable sensor interfaces in the literature [129,130]. In addition, prolonged skin contact under occlusive conditions may promote microbial colonization and biofilm development if surface properties are not carefully controlled. These challenges highlight the importance of antifouling surface coatings, material selection, and controlled wear duration when designing MN-based wearable platforms for long-term operation [129,130].

Recent studies highlight the promise of 3D-printed and flexible MNA electrodes. Liu et al. [131] have developed a 32-channel MNA dry electrode patch that was capable of recording electroencephalography (EEG), electrocardiography (ECG), and electromyography (EMG) signals with markedly reduced impedance compared with conventional electrodes. Li et al. [132] have further advanced this concept by developing a high-performance flexible MNA electrode for polysomnography and wearable electrophysiology, demonstrating superior stability and comfort during sleep studies. Juillard et al. [133] have developed hydrogel-based biodegradable MNAs to enable safe and long-term electrophysiological monitoring, offering new strategies that are suitable for transient or even pediatric applications.

Parrilla et al. [127] have demonstrated a wearable 3D-printed solid MNA voltammetric sensor for real-time uric acid detection in interstitial fluid. The device integrated three gold-coated MNA electrodes, working, reference, and counter, that were fabricated through stereolithographic 3D printing and metal sputtering. The nanostructured gold surfaces significantly enhanced the electrocatalytic activity, achieving high sensitivity (25 nA μM^−1^) and a wide linear range (150–500 μM) that is relevant for physiological uric acid levels. The sensor exhibited excellent reproducibility, good selectivity against common interferents (glucose, ascorbic acid, urea), and also mechanical robustness for repeated skin insertions.

Recent studies have demonstrated the feasibility of MNA-based electrochemical glucose sensing in real or biologically relevant samples. Zhou et al. [128] employed solid 3D-printed MNAs for in situ amperometric monitoring of ISF glucose, achieving high sensitivity and strong correlation with reference glucose levels in vivo. In contrast, Zhan et al. [115] utilized hollow MNAs combined with negative-pressure ISF extraction and patterned electrodes, enabling reliable glucose detection with minimal tissue damage but introducing additional system complexity. Also, in a recent study, Yang et al. [114] integrated MNAs into a wearable dual-sensor platform for continuous in vivo glucose monitoring alongside pharmacological biomarkers, demonstrating translational potential for personalized diabetes management. These studies highlight trade-offs between sensing accuracy, system integration, and long-term wearability. Also, a comparative summary of sensitivity, validation approach, and reported variability for representative MNA-based electrochemical glucose sensors evaluated in real samples is provided in Table 4.

**Figure 4:**
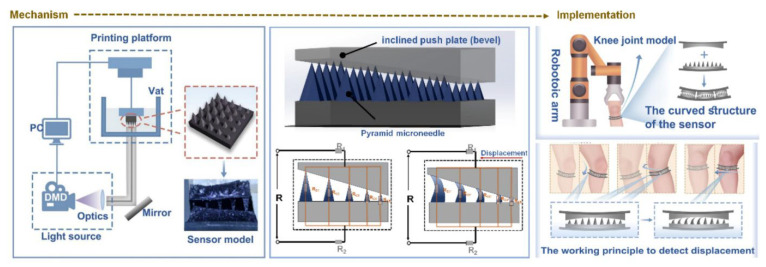
MNA-based sensor for motion monitoring. The system integrates hydrogel MNAs with an inclined push plate, which enables synchronized deformation and contact-area modulation to amplify displacement signals during micromotion detection. This design markedly improves sensitivity for capturing the subtle surface displacements that happen around the knee joint. The motion from the knee joint model is accurately controlled by using a six-degree-of-freedom robotic arm. Dashed boxes indicate highlighted/zoomed subviews of the device and sensing elements, and arrows denote the workflow/relationships between panels as well as the direction of applied loading/deformation and the resulting displacement/response.Reprinted under a CC BY license [111].

In another study, Zhang et al. [111] have reported a 3D-printed MNA-based wearable sensor that was capable of high-sensitivity detection of human joint motion. The device integrated a hydrogel MNA with a flexible conductive substrate, enabling it to sense the subtle mechanical deformations that are generated during knee flexion, extension, torsion, and also lateral movements. Figure 4 shows their designed 3D-printed MNA-based wearable motion sensor that couples structural precision with skin-conforming flexibility. The schematic outlines the complete workflow, from MNA fabrication via stereolithographic 3D printing to integration with hydrogel-based conductive substrate. When the patch is adhered on the skin, the mechanical deformation that is generated by joint movements (such as flexion or extension) is transmitted through the microneedle tips to the underlying sensing layer, producing a proportional electrical resistance change. The figure also depicts how the optimized microneedle geometry enhances strain transfer efficiency and adhesion, ensuring accurate signal capture even under dynamic motion. This mechanism enables real-time biomechanical monitoring with high sensitivity and minimal discomfort, demonstrating how polymeric MNA-integrated wearables can serve as reliable human–machine interfaces for neuromuscular diagnostics, rehabilitation feedback, and personalized movement tracking [111].

Recent advances further highlight the advantages of wearable microneedle-based devices for continuous and physiologically relevant monitoring. For example, Wei et al. [134] developed a wearable 3D-printed hollow microneedle temperature sensor capable of real-time intradermal monitoring with high sensitivity and stability. Parrilla et al. [135] reported a low-cost wearable patch based on 3D-printed solid microneedle arrays functionalized with nanostructured gold electrodes, enabling reliable in situ electrochemical monitoring of interstitial fluid biomarkers with minimal signal degradation after repeated skin insertion and on-body testing. More broadly, recent reviews of wearable microfluidic and microneedle-enabled systems emphasize their advantages in skin conformity, continuous data acquisition, and integration with wireless and AI-assisted platforms [136].

Beyond glucose and lactate monitoring, emerging MNA-based biosensing platforms are increasingly targeting a broader range of biomarkers, including electrolytes (e.g., Na^+^, K^+^), inflammatory cytokines, and drug metabolites in interstitial fluid [137]. While many MNA-based wearable systems demonstrate promising biomedical applications, clinical translation remains challenged by physiological and usage-related factors. Variations in skin properties, sweat accumulation, and body motion can influence insertion consistency, signal stability, and long-term performance, underscoring the need for robust device design and user-adaptive calibration strategies in real-world settings.

Potential failure scenarios in MNA-based wearable systems include needle fracture during insertion, incomplete penetration due to insufficient mechanical strength or skin variability, and delamination at rigid–soft interfaces under repeated motion. Mitigation strategies include optimizing needle geometry and aspect ratio, selecting materials with adequate fracture toughness, reinforcing base structures, and employing robust interfacial adhesion or elastomeric interlayers. Incorporating these design considerations is critical for ensuring safe and reliable long-term wearable operation.

In wearable electrophysiology, dry MNA electrodes and hydrogel-based MNA electrodes exhibit distinct performance trade-offs. Dry MNA electrodes typically offer lower contact impedance than conventional dry electrodes due to direct skin penetration but may experience higher impedance variability and motion sensitivity over time. In contrast, hydrogel-integrated MNAs generally provide more stable electrode–skin contact, reduced impedance drift, and improved signal-to-noise ratio (SNR), albeit with increased hydration dependence and potential long-term dehydration effects [138].

### 4.2. Drug Delivery

MNA-assisted drug delivery has been emerging as a transformative platform that is bridging the gap between conventional hypodermic injections and topical administration [44]. By forming transient microchannels without stimulating dermal nerves or blood vessels, MNAs significantly improve patient compliance and allow for self-administration, key attributes for next-generation wearable therapeutics. The integration of MNAs into wearable systems introduces new opportunities for the delivery of drugs, vaccines, and biomolecules, especially when coupled with microfluidic reservoirs or electronic sensors for dosage regulation.

Among the various types of MNAs, hollow MNAs represent a particularly promising design for wearable transdermal therapy. Their internal lumens permit the active pumping or diffusion of drug solutions from a reservoir into the skin, mimicking the functionality of a miniature syringe while avoiding pain and infection risk. This type of MNA can accommodate large molecules such as peptides, proteins, and nucleic acids that are otherwise impermeable through the skin [139]. The advent of 3D-printing technologies has been revolutionizing the design and manufacturability of hollow and polymeric MNAs, allowing precise control of geometry, internal channels, and also mechanical strength [140].

MNA-based drug-delivery systems can be broadly classified according to their primary transport mechanism. Diffusion-driven systems rely on passive drug release from dissolving or hydrogel-forming microneedles into the surrounding tissue. Pressure-driven systems employ applied mechanical forces or reservoirs to actively push a drug through hollow MN lumens. Iontophoretically assisted systems use a small electrical current to enhance drug flux across the skin via electromigration and electroosmotic flow [141].

**Figure 5:**
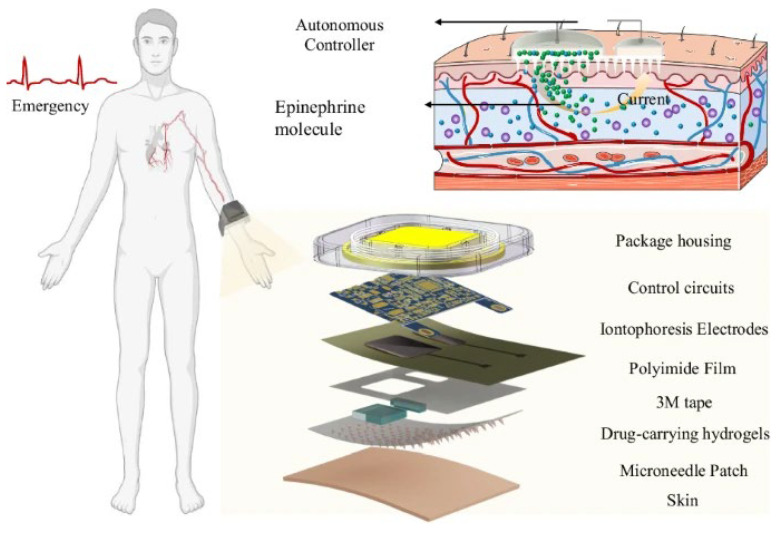
The conceptual design and operational principle of a wearable self-aid MNA chip for the active transdermal delivery of epinephrine. Colors are used for schematic clarity: red/blue lines indicate blood vessels, green dots represent the drug molecules, purple dots represent ions in the interstitial fluid, and the yellow arrow indicates the iontophoretic current path; the shaded regions denote skin/tissue layers and device components. Reproduced under a CC BY license [142].

Drug delivery using MNA-integrated wearable devices has been investigated in several different studies. For example, Jin et al. [142] have developed a wearable self-aid MNA-integrated device for the transdermal delivery of epinephrine, illustrating how integrating MNAs with electronic actuation can transform conventional drug delivery into an intelligent and self-regulated process. They have developed a wearable patch combining hydrogel-based MNAs, conductive materials, and an iontophoretic control system to enable the active and adjustable transdermal administration of epinephrine. Unlike passive MNA patches that rely on diffusion or dissolution, this design allowed users to regulate the dosage in real time through electronic control, representing a significant step toward more personalized and responsive pharmacotherapy. The research has demonstrated that active MNA systems can achieve the rapid onset of the drug action, comparable with injections, yet with improved comfort, safety, and user autonomy. The concept of the “press-to-deliver” operation is making it possible to use such devices for emergency self-aid, particularly in situations where immediate medical assistance is not available. Figure 5 schematically illustrates their developed wearable self-aid MNA-based device. The figure shows the multilayer structure of the device, consisting of hydrogel MNAs, conductive interfaces, and an iontophoretic circuit that enables electronically controlled and on-demand drug delivery. The schematic also depicts the user-activated operation and the emergency self-aid application scenario [142].

For electrically assisted epinephrine delivery, safety considerations related to current exposure are critical. Wearable iontophoretic or electrically actuated systems typically operate at low current densities designed to remain below sensory and tissue damage thresholds; however, prolonged exposure, skin condition variability, and electrode placement can influence local irritation or discomfort. Careful current control, fail-safe circuit design, and compliance with established safety standards are therefore essential for emergency-use applications.

**Figure 6:**
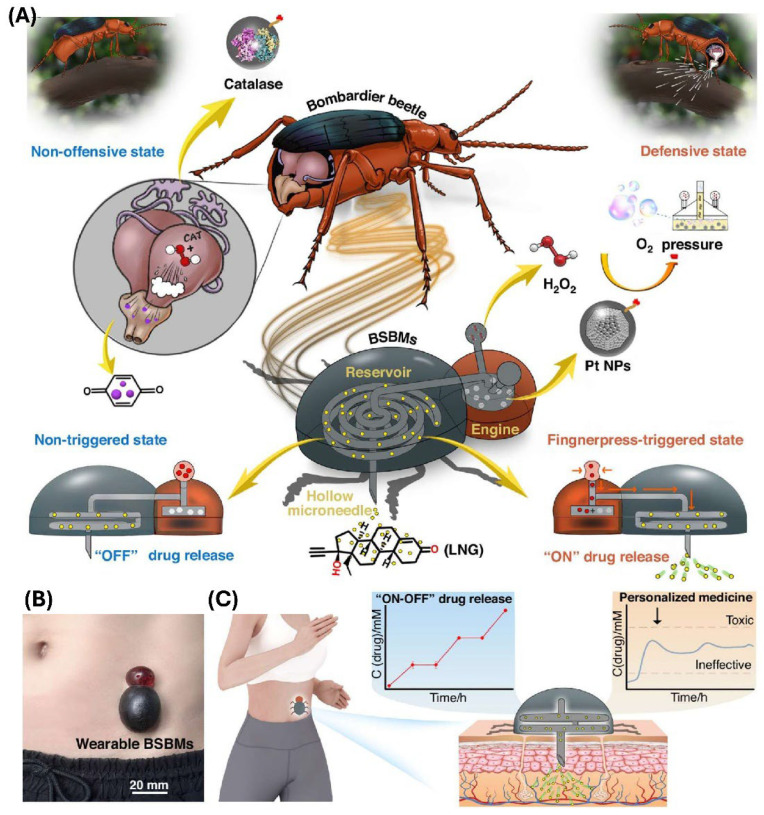
The design concept and operation of a battery-free and self-propelled bionic MNA drug-delivery system. (**A**) Schematic representation of bombardier beetle-inspired self-propelled biomimetic MNA system and multidimensional bionic strategies from material and structure to function of the bombardier beetle’s defensive discharge secretion mechanism. Blue/orange labels denote OFF (non-triggered)/ON states; yellow arrows show process/transport flow; green dots indicate released drug; O_2_/pressure icons indicate pressurization, (**B**) Image of the self-propelled biomimetic MNA system adhered to the skin, (**C**) Wearable self-propelled biomimetic MNA system for on-demand control of the level of levonorgestrel within the medication window range. Green dots indicate drug delivered into skin Reproduced under a CC BY license [143].

In a study, Tan et al. [143] have developed a battery-free and self-propelled bionic MNA system that enables drug delivery without any external power sources. Inspired by the bombardier beetle’s defensive spray mechanism, the device integrated a miniature “bionic engine,” a drug reservoir, and a hollow MNA into a compact 3D-printed patch. The propulsion force arose from a catalytic reaction between platinum nanoparticles and hydrogen peroxide, generating oxygen pressure that actively drove the drug through the MNA into the skin. This design eliminates the need for pumps, wiring, or batteries while still allowing precise manual activation through a simple finger press. In vivo pharmacokinetic studies on rats using levonorgestrel as a model contraceptive drug have revealed controlled release that maintained therapeutic plasma levels within the desired range for extended durations. The drug absorption was proportional to the dosage and was sustained up to 54 h after repeated administrations. Histological and cytocompatibility assessments have confirmed minimal inflammation, high cell viability, and negligible tissue irritation, supporting excellent biocompatibility. Figure 6 illustrates their developed bioinspired propulsion principle that is based on catalytic gas generation, structural integration between the bionic engine and the hollow MNA, and the representative results that show controlled and repeatable drug release without any external power [143].

For bionic propulsion systems based on catalytic decomposition of hydrogen peroxide, chemical safety is a critical consideration. Although low-concentration hydrogen peroxide can be handled safely in controlled microfluidic environments, risks related to local tissue irritation, oxidative damage, and uncontrolled gas generation must be carefully managed. In addition, potential leaching of catalytic materials or residues raises biocompatibility concerns.

### 4.3. Painless Vaccination Platforms

The COVID-19 pandemic has brought a renewed urgency regarding the development of alternative vaccination technologies. For example, dissolvable MNAs could be utilized for COVID-19 immunization, highlighting their ability to deliver vaccines into the epidermis and dermis that are rich in antigen-presenting cells, thereby enhancing immunogenicity when compared with intramuscular injections [144]. Research has shown the benefits of MNA-based delivery, including the avoidance of cold-chain storage, reduced medical waste, and also the possibility for large-scale distribution without the need for professional administration. This paradigm is especially relevant for rapid-response vaccination campaigns during pandemics.

Studies have shown the versatility of MNAs in the transdermal delivery of biopharmaceuticals, including vaccines, therapeutic proteins, and nucleic acids [145]. MNA-mediated immunization offers both dose-sparing effects and enhanced patient acceptance due to its pain-free administration. Advances in 3D printing and polymeric MNA fabrication enable precise control of geometry, mechanical strength, and drug-loading efficiency, which are critical parameters for vaccine delivery patches. Importantly, the use of e biodegradable polymers allows the needles to dissolve safely within the skin, eliminating sharps waste and lowering infection risks.

MNA-based vaccination has been shown to elicit immunogenic responses comparable to or greater than intramuscular injection while enabling significant dose sparing. By delivering antigens into the epidermal and dermal layers rich in antigen-presenting cells, MNAs can achieve equivalent antibody and cellular immune responses using substantially lower antigen doses, highlighting the immunological advantage of skin-targeted vaccination [146].

The integration of wearable technologies is further amplifying the potential of MNA vaccination systems. Research has presented a wearable and flexible ultrasound MNA for cancer immunotherapy, showing how dissolvable MNAs that are combined with soft and skin-conformal electronics can achieve robust adhesion, painless drug delivery, and localized immune modulation [125]. While this work focused on oncology, its design principles, including flexible substrates, bioadhesive interfaces, and minimally invasive MNA insertion, are directly applicable to vaccination wearables. By coupling antigen-loaded polymeric MNA with wearable platforms, painless vaccination patches can be developed to provide sustained immune stimulation, potentially coupled with biosensing and feedback control for precision immunization.

Beyond conventional antigen-based vaccines, MNAs have been explored for nucleic acid delivery to the skin, as demonstrated by transdermal small interfering RNA administration using solid microneedle arrays. Deng et al. showed that microneedle-mediated delivery enabled efficient penetration of cholesterol-modified small interfering RNA into the epidermis, resulting in localized gene silencing of up to 66% without significant accumulation in major organs [147].

Together, these advances are highlighting the growing feasibility of MNA-integrated wearables as painless vaccination platforms. By combining the precision of 3D-printed polymeric MNAs with dissolvable materials and wearable electronics, future vaccine delivery systems could achieve unprecedented accessibility, safety, and also patient compliance. Such platforms are not only promising to overcome needle-associated barriers but also offer a scalable and sustainable approach for global immunization efforts.

### 4.4. Personalized and On-Demand Drug Delivery

MNA-integrated wearables are emerging as powerful tools for personalized and on-demand drug delivery, addressing the limitations of conventional transdermal and systemic administrations. A central innovation in this domain is the development of remote-controlled MNA systems. A hollow 3D-printed MNA platform that was capable of both biosensing and drug delivery, activated by external stimuli for on-demand release, has been developed by our group [26]. By embedding colorimetric sensors and enabling wireless actuation, this system demonstrated how MNAs can transition from static drug depots into dynamic therapeutic devices that are tailored to an individual’s physiological state.

Innovations in multifunctional MNAs are also enabling simultaneous biosensing and therapeutic interventions. MNAs are capable of sensing, extracting fluids, and delivering drugs, underscoring the need for materials and fabrication techniques that support multiplex operations. There are challenges in achieving stable performance, material biocompatibility, and robust adhesion in wearable contexts, but it is underscored that multifunctionality is critical to advance on-demand therapies [112].

The foundation for such strategies has been established by Economidou et al. [54], who have developed a 3D-printed hollow MNA MEMS that was designed for the controlled and personalized delivery of small molecules. This work highlighted the capacity of additive manufacturing to produce customized needle geometries and channels, optimizing drug-delivery profiles based on patient-specific requirements. Such devices can deliver precise doses directly into the dermis, where the high vascularization ensures rapid absorption.

Stimuli-responsive MNAs represent another important class of on-demand platforms. Smart polymers that are responsive to pH, temperature, reactive oxygen species (ROS), or light enable the triggered release of therapeutics at targeted sites [32]. For example, pH-responsive MNAs can selectively release anti-inflammatory drugs in diseased tissues, while light-responsive systems enable precise spatiotemporal control. These approaches are particularly promising for chronic diseases that require adaptive dosing strategies.

Wearable MNAs are also being combined with self-powered systems to enhance autonomy. For example, a hydration monitoring and drug-delivery patch has been developed that harvests biomechanical energy to sustain closed-loop treatment for atopic dermatitis [120]. This design eliminates reliance on external power sources and provides responsive therapy that is aligned with patient-specific hydration levels. Such innovations are paving the way toward personalized and long-term management for dermatological and metabolic disorders.

Wu et al. [148] have introduced a wearable acoustic AMNA platform for on-demand and programmable transdermal drug delivery. The system integrated 3D-printed hollow MNAs with a miniaturized piezoelectric transducer that was controlled through a Bluetooth-enabled smartphone app. The acoustic waves generated localized microvortices at the needle tips, actively pumping liquid drugs through the skin with precise dosage control. The device supported single, batch, and continuous release modes, enabling personalized treatment scheduling. In vivo mouse studies have demonstrated accurate dosage regulation, good biocompatibility, and rapid skin recovery, showcasing a next-generation approach to smart and user-controlled therapeutic delivery for both chronic and acute disease management.

Closed-loop MNA-based delivery systems, like the integrated addressable MNA for glucose monitoring and insulin release, primarily use threshold-based triggering with rule-based logic. Real-time glucose sensing data is sent wirelessly to a mobile app, which alerts the user and activates insulin release from selected microneedles via reductive voltage when glucose exceeds set thresholds. This design emphasizes simplicity, low power use, and reliability (e.g., redundant sensors), while avoiding complex passive mechanisms. It may require user confirmation and could cause oscillations if thresholds are poorly tuned. Advanced methods like proportional–integral–derivative (PID) or model predictive control, common in traditional closed-loop insulin systems, are avoided due to higher computational, power, and regulatory demands in miniaturized devices [149,150,151].

**Figure 7:**
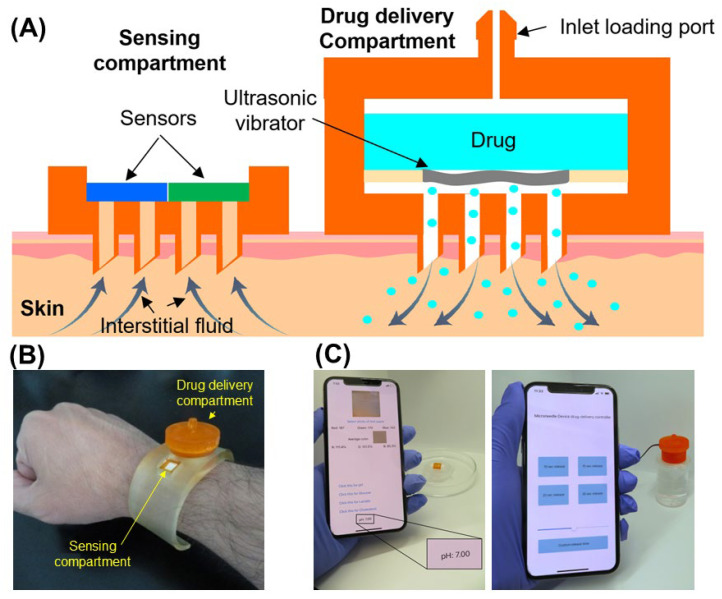
An MNA-based theranostic platform. (**A**) The concept of the developed device. Arrows indicate the direction of interstitial fluid/analyte transport toward the sensing MNA and the diffusion/transport of the released drug from the delivery MNA into the skin; (**B**) developed wearable device; (**C**) smartphone control enabled sensing and drug delivery. Reproduced under a CC BY license [26].

In another study, Razzaghi et al. [26] have developed a wearable theranostic platform that integrated colorimetric sensing and on-demand drug delivery by using 3D-printed hollow MNAs. The device combined a biosensing compartment for quantifying pH, glucose, and lactate with a remotely triggered ultrasonic atomizer that enabled wireless and controlled drug administration. Mechanical tests have confirmed MNAs’ structural integrity and consistent penetration force, while ex vivo experiments on pig skin have verified uniform dye diffusion, confirming reliable tissue insertion. The system demonstrated precise colorimetric sensing across physiologically relevant ranges using a smartphone-based application for real-time analysis. Ultrasonic-assisted drug delivery significantly enhanced diffusion through hydrogel models compared with topical or passive MNA delivery, and the modulation of the ON–OFF cycles validated controllable dosing profiles. This study demonstrated a multifunctional and smartphone-controlled MNA that was capable of both non-invasive biochemical monitoring and wireless on-demand drug delivery, marking a major step toward closed-loop wearable therapeutics. Figure 7 depicts their 3D-printed MNA-integrated system. This integrated system exemplifies a self-contained wearable patch that is capable of real-time sensing and programmable therapeutic administration [26].

**Figure 8:**
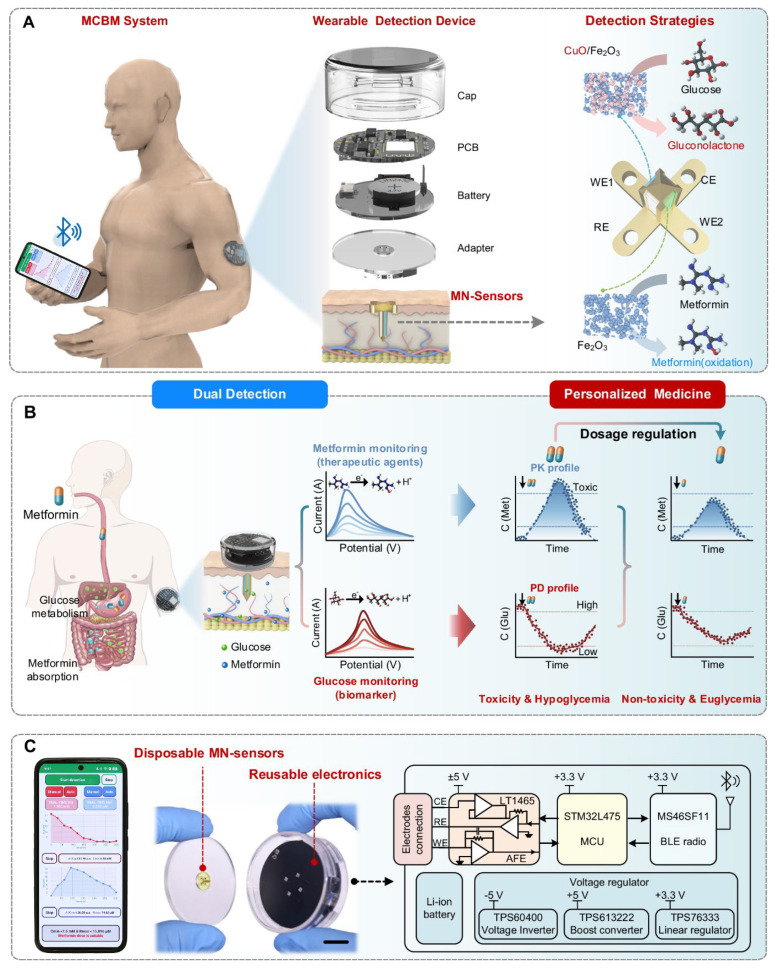
(**A**) Schematic representation from the self-developed microneedle-based continuous biomarker monitoring (MCBM) system, comprising the MCBM device together with a dedicated smartphone application. The exploded view illustrates the detection mechanisms for both glucose and metformin sensors. (**B**) The MNA-based minimally invasive dual-biomarker sensor that is designed for the precise monitoring of glucose and metformin levels, supporting personalized medicine through pharmacokinetic/pharmacodynamic (PK/PD) assessment and feedback-controlled therapy. (**C**) Photographs of the fully integrated smartphone-assisted wearable platform, highlighting the two modular components from the MCBM device, disposable and reusable, alongside a block diagram detailing the wearable electronic system. Scale bar: 1 cm. Reprinted under a CC BY license [114].

In another study, Yang et al. [114] have developed an MNA-based continuous biomarker/drug monitoring (MCBM) system that unifies pharmacokinetic and pharmacodynamic assessments for personalized diabetes therapy. The platform integrated dual microneedle electrochemical sensors that were capable of simultaneously detecting metformin and glucose concentrations in interstitial fluid (ISF). Each microneedle was functionalized with a nanoenzyme-modified electrode, ensuring high selectivity, sensitivity, and stability under physiological conditions. The real-time ISF analysis was wirelessly transmitted to a smartphone interface, which computed metabolic feedback to inform dynamic dose adjustment. In in vivo experiments, an MCBM patch has achieved a strong correlation with blood glucose profiles and pharmacological dosing curves, demonstrating reliable pharmacokinetic–pharmacodynamic (PK–PD) coupling during metformin administration. This closed-loop sensing framework highlights how MNA-integrated wearables can transcend single-parameter monitoring to provide continuous therapeutic feedback and precise dosage control that is tailored to individual physiology. The study represents an important step toward next-generation intelligent drug-delivery systems, merging biochemical sensing, data analytics, and wearable connectivity to enable truly smart, adaptive, and personalized healthcare management. Figure 8 shows their designed integrated MNA-based PK–PD monitoring system that enables real-time and personalized drug management. The schematic illustrates the overall architecture of the MNA-based continuous biomarker/drug monitoring (MCBM) platform, which combines dual electrochemical microneedle sensors for the simultaneous detection of metformin and glucose in the interstitial fluid. The system couples these biochemical measurements with wireless data transmission to a smartphone interface, where customized algorithms process the collected signals to map both drug kinetics and glucose response profiles. This integrated feedback loop allows the dynamic adjustment of therapeutic dosing based on the patient’s metabolic state. The figure also highlights the device’s wearable form factor, 3D-engineered MNAs, and nanoenzyme-modified electrodes, which collectively ensure stable signal acquisition and high analytical sensitivity. Overall, the figure effectively visualizes how MNA-integrated wearable systems can unify biosensing, pharmacological modeling, and wireless communication into a closed-loop framework for personalized and adaptive therapy [114].

Compared to conventional hypodermic injections, the drug-delivery capacity of MNA systems strongly depends on their structural design. Solid, coated, and dissolving MNAs are inherently limited in drug dose, as the payload is restricted to the needle surface or internal matrix, typically confining delivery to microgram or sub-milligram levels. In contrast, hollow MNAs enable fluid-based delivery through the internal lumens and can be coupled with external reservoirs, allowing substantially higher drug volumes and continuous or on-demand administration [139]. Recent advances in 3D-printed hollow MNAs integrated with refillable reservoirs and active delivery mechanisms have demonstrated drug-delivery performance approaching that of hypodermic injections while maintaining minimal invasiveness and improved patient compliance [26]. These developments position hollow MNAs as a scalable alternative for applications requiring higher therapeutic doses. Table 5 presents an overview of wearable MNA platforms, applications, and key outcomes.

## 5. Challenges and Future Perspectives

One of the main challenges in developing 3D-printed MNA-integrated wearable devices is the regulatory issues. More specifically, the regulatory pathways for 3D-printed MNAs as medical devices or drug-delivery tools are quite complex. The dual nature of MNAs, being both a device and a drug-delivery system, complicates approval processes, requiring compliance with multiple sets of regulations [82,152]. Regulatory approval often requires a demonstration of consistent and safe penetration depths avoiding reaching the dermal vasculature or the nerves. Studies like those by Defelippi et al. have focused on depth control, but further standardization is still needed [153]. Currently, approvals for MNAs are decided on a case-by-case basis rather than through a unified regulatory system, leading to prolonged licensing processes and slowing down market entry. To solve this issue, a comprehensive regulatory framework is needed to cover key aspects like device shape, formulation, sterilization, and also packaging. Integrating the current Good Manufacturing Practice (cGMP) guidelines and applying a quality-by-design approach would facilitate the approval process and improve the reliability of MNAs as pharmaceutical products [154].

From a regulatory perspective, MNA-based systems are classified based on intended function and integration. In the United States, devices for aesthetic skin penetration alone are Class II medical devices (21 CFR 878.4430, requiring 510(k) clearance). Drug-delivery MNAs (e.g., coated or dissolving) are typically combination products (drug–device), with the primary mode of action (PMOA) determining the lead center and requiring coordinated FDA review. In the European Union, classification depends on the principal mode of action under MDR (EU 2017/745). Device-driven systems (e.g., mechanical penetration) are regulated as medical devices; integral drug–device combinations where the drug is principal are treated as medicinal products, with the device component conforming to MDR General Safety and Performance Requirements, often via Notified Body involvement [155,156,157].

In addition to regulatory challenges, the development of MNAs is also facing significant challenges in patenting and intellectual property. Numerous patents that are related to 3D-printed MNAs have already been issued [158,159,160]. The increasing number of patents in this field, which are predominantly held by industry, highlight the competitive nature of innovation. Overly broad or restrictive patents can hinder technological progress by limiting competition and accessibility. With patents covering diverse aspects such as drug delivery, fabrication methods, and device applications, securing freedom to operate is becoming quite complex. Additionally, overlapping claims and the broad Cooperative Patent Classification (CPC) system create legal uncertainties, which can potentially slow commercialization. Future advancements require clearer regulatory frameworks and collaborative licensing models to balance innovation with accessibility and to ensure sustainable development of MNA technology [161]. Additionally, strengthening collaboration between regulatory agencies, industry stakeholders, and patent offices can help to establish clearer guidelines for MNA patenting while balancing innovation together with market accessibility.

Environmental and on-body factors can significantly influence the performance of wearable MNA systems. Variations in humidity and temperature may affect polymer swelling, drug release kinetics, and sensor stability, while sweat accumulation and body hair can interfere with skin contact, adhesion, and signal reliability. Addressing these factors through material selection, surface engineering, and adaptive device design is essential for robust real-world deployment.

The future development of the 3D-printed MNAs for wearable devices is facing significant challenges in clinical translation and trials. Despite encouraging laboratory findings, translating these innovations into clinical practice has been quite a challenge. The limited availability of long-term clinical data remains a major hurdle for regulatory approval and widespread adoption. While there has been an increase in clinical studies, with a shift from early-stage trials (Phases 1 and 2) toward later stages (Phases 3 and 4), a large proportion of trials remain unclassified, indicating regulatory uncertainty. Many trials focus on drug delivery, mainly targeting skin conditions, vaccines, and also ocular treatments, but there is still a lack of large-scale trials to establish long-term safety and also efficacy. Additionally, industry-sponsored trials are fewer than academic studies, which can potentially delay commercialization. Overcoming these hurdles will require a more coordinated effort between academia, industry, and regulatory agencies to streamline approvals and accelerate clinical adoption [161,162].

Another major challenge in the future development of 3D-printed MNAs for wearable devices is scalability. Although 3D-printed MNAs offer remarkable potential, expanding their production for widespread clinical applications remains a major challenge. The process of 3D printing depends on specialized equipment and materials, resulting in high production costs that hinder large-scale manufacturing. For instance, high-resolution 3D printers are often too slow and expensive for commercial applications. Also, scalability depends on maintaining consistent quality as production volumes increase. Ensuring uniformity in the dimensions, tip sharpness, and material properties across batches is crucial to guarantee both safety and effectiveness. Another significant obstacle is the lack of standardized manufacturing methods and the inherent complexities of production. Many developers are still relying on manual, lab-scale fabrication methods, which are not suitable for mass production. The wide variation among MNA designs, formulations, and also applications necessitates the development of specialized equipment and innovative manufacturing processes [163]. Addressing these scalability challenges is critical to transition MNAs from research prototypes to commercially viable medical devices that can be integrated into broader healthcare markets.

The choice of materials significantly impacts the mechanical strength and biocompatibility of MNAs. Polymers often lack the mechanical robustness that is required for effective skin penetration without breakage [164]. Metals and ceramics offer higher strength but are harder to fabricate with high precision by using 3D-printing techniques [165,166]. Also, the fabrication of MNAs presents several technical difficulties. Achieving the desired geometry, especially the inner diameter (ID) and the outer diameter (OD) of the needles, is quite challenging. Even high-resolution 3D-printing techniques are facing some limitations in fabricating extremely fine and uniform bore sizes [152,165,166]. Despite the advancements in 3D printing, several challenges persist in 3D printing MNAs. Consistent lumen formation remains a significant hurdle, since even minor defects can compromise fluid flow. Material biocompatibility is another critical issue, especially for long-term applications.

Scaling up the production of MNAs while maintaining cost-effectiveness is also a significant hurdle. Three-dimensional printing offers customization and precision but at a high cost per unit compared with traditional manufacturing methods like molding. This is especially problematic for applications that require large-scale deployment [43,61]. The fabrication of MNAs requires specialized equipment and skilled personnel, adding to the overall cost. Training and retaining a workforce that is proficient in 3D-printing technologies is still a bottleneck in many regions [152,165].

Maintaining uniformity across batches is also challenging because of the sensitivity of 3D printing processes to parameters like temperature, resin composition, and printing speed. Such variability can lead to inconsistencies in needle dimensions and drug-delivery performance [152,164]. Table 6 provides a consolidated overview of the key design strategies, materials, fabrication methods, and applications discussed in this review.

## 6. Conclusions

The integration of polymeric 3D-printed MNAs with wearable devices represents a transformative advancement in the evolution of personalized and connected healthcare. Through the convergence between additive manufacturing, smart materials, and flexible electronics, these hybrid systems are redefining how physiological data is acquired, processed, and acted upon in real time. Unlike conventional wearables that mainly monitor superficial signals, MNA-based platforms penetrate through the skin’s outer layer to access the interstitial fluid (ISF), which is a rich biochemical medium that mirrors the blood composition, enabling minimally invasive monitoring, precise drug delivery, and continuous therapeutic feedback. This unique capability positions MNA wearables as a cornerstone technology for next-generation digital health systems that are aiming to merge diagnostics, therapy, and decision support within a single skin-conformal patch.

The recent progress in 3D printing has been instrumental for advancing MNA functionality and manufacturability. High-resolution techniques such as SLA, DLP, and 2PP allow the fabrication of hollow, solid, and hybrid MNAs with tunable geometry, lumen diameter, and surface topography. These capabilities surpass the limitations of conventional molding or lithographic methods, providing superior precision, good reproducibility, and also design flexibility. The use of polymeric materials like PEGDA, PLA, PCL, and the GelMA has been further expanding the performance window of MNAs, offering the biocompatibility, mechanical compliance, and stimuli-responsive behavior that are essential to achieve proper skin integration and adaptive functionality. Through material and process optimization, researchers have been demonstrating MNA systems that can dissolve, swell, conduct electricity, or release therapeutics on demand, all within a lightweight and wearable configuration.

The synergistic integration between MNAs, flexible electronics, and microfluidic systems has been propelling the development of skin-conformal platforms that are capable of multiplexed sensing and closed-loop control. Advances in wireless communication, power harvesting, and embedded computing are now allowing these devices to operate as autonomous “wearable labs,” continuously sampling ISF, transmitting biochemical data, and triggering therapeutic responses without any external intervention. Notable demonstrations include glucose sensing and insulin releasing patches, MNA vaccination platforms, and self-powered drug-delivery systems. Such a convergence between sensing, actuation, and data analytics is marking a paradigm shift toward real-time and patient-centric healthcare, where continuous monitoring is informing precise and adaptive interventions that are tailored for individual needs.

Despite this rapid progress, several key challenges still need to be addressed before a widespread clinical translation can really occur. Manufacturing scalability remains a major hurdle and, while 3D printing is allowing unmatched customization, its throughput and cost efficiency are still lagging behind that of traditional mass-production techniques. Standardized fabrication protocols, post-processing methods, and quality control measures are essential to ensure reproducibility and safety across production batches. Regulatory uncertainty also persists because of dual classification of MNAs as both medical devices and drug-delivery systems. Establishing unified frameworks that encompass sterility, biocompatibility, mechanical reliability, and long-term skin compatibility will be crucial for commercial adoption. In addition, maintaining the stability of biological recognition elements (e.g., enzymes, antibodies) and preventing biofouling on the skin interface remain as technical challenges that must be overcome to ensure durable sensor performance.

Looking ahead, the field is poised to benefit from emerging innovations that are happening at the interface between materials science, bioelectronics, and data intelligence. The integration of AI and machine learning into MNA-based wearables will enable predictive analytics and adaptive control for therapeutic regimens. Self-powered systems that use piezoelectric, triboelectric, or thermoelectric energy harvesters could eliminate the dependence on external batteries, enhancing both autonomy and comfort. Furthermore, advances in bioprinting and multimaterial 3D printing will facilitate the fabrication of hierarchical microneedle structures that incorporate living cells, hydrogels, and conductive networks for advanced sensing and tissue-interactive applications. Cloud connectivity, cybersecurity, and user-centric interface design will further determine the success of these technologies in real-world healthcare environments.

In summary, polymeric 3D-printed MNA wearables are emerging as highly promising platforms that are uniting precise microfabrication, intelligent materials, and digital health analytics into a single system. They are bridging the gap between invasive diagnostics and non-invasive monitoring, offering continuous access to molecular information and controlled therapeutic delivery with minimal discomfort. Multidisciplinary collaboration among engineers, material scientists, clinicians, and regulatory bodies will be essential to transform the current laboratory prototypes into safe, manufacturable, and clinically validated products. With sustained innovation and standardization, MNA-integrated wearables are poised to redefine personalized healthcare, transitioning medicine from reactive treatment toward proactive, autonomous, and data-driven well-being.

## Figures and Tables

**Table 1:** An overview of MNA types, technologies, costs, and application fields.

MNA Type	Key Technologies	Cost Insights	Primary Applications	Key Specifications
Solid	SLA, DLP, LCD	Low for prototyping; higher for high-res	Biosensing (e.g., glucose, motion)	High signal stability; minimal drift in wearables
Hollow	DLP, 2PP, FDM	Moderate; scalable with LCD	Drug delivery, vaccination	Controlled flow; comparable to injections without pain
Dissolving	SLA, FDM	Cost-effective for mass prod	Sustained release, cosmetics	Dose-sparing immunity; reduced waste
Coated	SLA, DLP	Variable; post-processing adds cost	Targeted biologics delivery	Enhanced bioavailability; painless administration
Hydrogel-Forming	DLP, 2PP	Higher due to material tunability	Continuous sensing, therapy	Swelling enables sustained sampling; minimal irritation

**Table 2:** A comparison of MNA 3D-printing techniques, materials, achievable dimensions, strengths, limitations, and TRLs.

Technique	Materials	Resolution	Strengths	Limitations	Estimated TRL
SLA	Liquid photopolymers (e.g., UV-curable resins, PEGDA, biocompatible Class I resins)	~25–100 μm	High precision for complex geometries; smooth surfaces; versatile for master molds in ocular/insulin delivery; enables transdermal electrochemical sensing and biomarker detection	Slower point-by-point curing; anisotropy and oxygen inhibition affect mechanical properties; cytotoxicity from residuals requires post-processing; high costs for advanced setups	5–7
DLP	Photopolymer resins (e.g., PEGDA, biocompatible light-sensitive resins)	~30–100 μm	Accelerated production vs. SLA; supports on-demand drug delivery and biomarker detection (pH, glucose, lactate); in vivo glucose monitoring; enhanced buccal permeability	Potential pixelation; similar biocompatibility issues as SLA; sensitivity to curing time affects strength; scalability challenges	5–7
LCD	Light-curable photopolymer resins	~30–100 μm	Cost-effective for large scale; higher speeds than SLA; minimal distortion; suitable for wearable prototypes with high accuracy	Longer exposure times than DLP; larger tip radii reduce penetration efficiency; reduced light intensity limits curing depth	4–6
SOPL	Monomer solutions for polymerization	~10–50 μm	Rapid, high-throughput without mechanical movement; superior mechanical strength vs. DLP; quick customization; minimizes tissue damage	Limited flexibility in pattern changes; less established for diverse applications	4–5
2PP	Photosensitive resins (e.g., PVP/PVA, acrylate-based, Ormocer^®^)	<1 μm	Submicron resolution for detailed structures; excellent control over barbs and arrays; supports stimuli-responsive designs; high-speed potential for scaling	Expensive equipment; time-consuming; limited material options; requires precise control to avoid damage	4–6
FDM	Thermoplastics (e.g., PLA, biodegradable polymers like ABS, PVA)	~50–200 μm	Economical and user-friendly; no molds needed; suitable for biodegradable prototypes and simple geometries	Low inherent resolution requires post-processing (e.g., etching causes roughness); heat degrades drugs; poor fidelity for complex MNAs	5–6

**Table 3:** Comparison of representative polymer materials used in microneedle arrays for wearables.

Polymer Type	Young’s Modulus (Typical)	Swelling Behavior	Degradation Profile	Suitability for Drug Loading
PVA/PVP	~0.1–2 GPa (dry)	High (water-soluble)	Rapid dissolution	Excellent for rapid drug release
PLGA	~1–3 GPa	Minimal	Hydrolytic, weeks–months	Good for sustained drug delivery
PEGDA hydrogels	~10 kPa–10 MPa (tunable)	High, tunable	Non-degradable or slow	Suitable for diffusion-controlled loading
GelMA	~10–100 kPa	Moderate–high	Enzymatic, days–weeks	Good for bioactive payloads
Photopolymer resins	~1–3 GPa	Low	Non-degradable	Limited; requires surface modification

**Table 4:** Summary of representative MNA-based electrochemical glucose sensing studies for real or biologically relevant samples.

Electrochemical Mode	Sample Type	Sensitivity (Reported)	Accuracy/Validation	Uncertainty/Variability	Study
Amperometric (enzymatic)	ISF	High sensitivity (linear response)	Strong correlation with reference glucose	Repeatability reported; explicit uncertainty not quantified	[128]
Amperometric (extracted ISF)	ISF	Enhanced signal via patterned electrodes	Reliable detection with minimal tissue damage	Variability discussed qualitatively	[115]
Electrochemical dual sensor	ISF	High sensitivity for continuous monitoring	Validated against pharmacokinetic profiles	Long-term stability demonstrated; uncertainty not explicitly reported	[114]

**Table 5:** Overview of Wearable MNA Platforms, Applications, and Key Outcomes.

MNA Type	Wearable Integration/Technology	Target Function	Key Results/Performance Outcomes
Solid MNAs	Dry electrodes on flexible substrates	Biosensing (ISF glucose)	High correlation with reference glucose values in vivo; stable amperometric response during continuous wear
Solid MNAs	Nanostructured electrodes + wearable patch	Electrochemical biosensing	Maintained signal stability after repeated skin insertions; low signal drift during on-body testing
Hydrogel-forming MNAs	Swellable MNAs with integrated electrodes	Continuous metabolite sensing	Enabled sustained ISF sampling with minimal skin irritation; improved signal stability over time
Hollow MNAs	Microfluidic channels + wearable reservoir	Drug delivery	Delivered drug volumes comparable to hypodermic injection; controlled flow rate under wearable operation
Dissolving MNAs	Polymer dissolution in skin	Vaccination	Achieved comparable or enhanced antibody titers with dose-sparing vs. intramuscular injection
MNAs + iontophoresis	MNAs with wearable electrodes and power source	On-demand drug delivery	Provided stable current densities (10–100 µA cm^−2^) enabling controlled transdermal delivery within safety limits
MNAs + sensors and actuators	Biosensors, wireless modules	Closed-loop therapy	Demonstrated real-time biomarker monitoring coupled with responsive drug delivery

**Table 6:** Summary of key design strategies, materials, fabrication methods, and applications of polymeric 3D-printed MNA-based wearable systems.

Design Strategy	Materials	Fabrication Method	Functional Integration	Primary Applications
Solid, dissolving, hydrogel-forming MNAs	Biodegradable polymers (PVA, PVP), hydrogels	SLA, DLP, molding via 3D-printed masters	Passive release, skin permeability enhancement	Vaccination, low-dose drug delivery, biosensing
Hollow MNAs (lumen-based)	Photopolymers, elastomers, composite resins	High-resolution SLA, DLP, 2PP	Reservoir-enabled delivery, microfluidics	High-dose drug delivery, insulin, biologics
Skin-conformal MNA wearables	Flexible polymers, conductive composites	Multimaterial 3D printing	Soft electronics, stretchable substrates	Continuous biosensing, wearable diagnostics
Actuated/controlled MNAs	Stimuli-responsive polymers	Advanced 3D printing + system integration	Acoustic, iontophoretic, or wireless control	On-demand and closed-loop therapeutics
Integrated smart MNA systems	Hybrid polymers, nano/biofunctional coatings	Multimaterial and high-resolution 3D printing	AI control, wireless communication	Personalized medicine, remote healthcare

## Data Availability

No new data were created or analyzed in this study. Data sharing is not applicable to this article.

## Abbreviations

The following abbreviations are used in this manuscript:

2PP	Two-Photon Polymerization
3D	Three-Dimensional
AI	Artificial Intelligence
cGMP	Current Good Manufacturing Practice
CPC	Cooperative Patent Classification
DLP	Digital Light Processing
ECG	Electrocardiography
EEG	Electroencephalography
EMG	Electromyography
FDM	Fused Deposition Modeling
FEA	Finite Element Analysis
GelMA	Gelatin Methacryloyl
GOD	Glucose Oxidase
ID	Inner Diameter
ISF	Interstitial Fluid
LCD	Liquid Crystal Display
LED	Light-Emitting Diode
MCBM	Microneedle-based Continuous Biomarker Monitoring
MEMS	Microelectromechanical System
MNA	Microneedle Array
NFC	Near-Field Communication
NIR	Near-Infrared
OD	Outer Diameter
PB	Prussian Blue
PCL	Poly(ε-caprolactone)
PEGDA	Poly(ethylene glycol) Diacrylate
PK–PD	Pharmacokinetics–Pharmacodynamics
PLA	Poly(lactic acid)
PVA	Poly(vinyl alcohol)
PVP	Poly(vinylpyrrolidone)
ROS	Reactive Oxygen Species
SLA	Stereolithography
SOPL	Static Optical Projection Lithography
UV	Ultraviolet
